# 5-Chloro-*N*-{4-oxo-2-[4-(trifluoro­meth­yl)phen­yl]-1,3-thia­zolidin-3-yl}-3-phenyl-1*H*-indole-2-carboxamide

**DOI:** 10.1107/S1600536812039347

**Published:** 2012-09-22

**Authors:** Mehmet Akkurt, İsmail Çelik, Füsun Kazan Gürbüzel, Sumru Özkırımlı, Orhan Büyükgüngör

**Affiliations:** a Department of Physics, Faculty of Sciences, Erciyes University, 38039 Kayseri, Turkey; b Department of Physics, Faculty of Arts and Sciences, Cumhuriyet University, 58140 Sivas, Turkey; c Department of Pharmaceutical Chemistry, Faculty of Pharmacy, Istanbul University, 34116 Beyazit, Istanbul, Turkey; d Department of Physics, Faculty of Arts and Sciences, Ondokuz Mayıs University, 55139 Samsun, Turkey

## Abstract

In the title compound, C_25_H_17_ClF_3_N_3_O_2_S, the five-membered 1,3-thia­zolidine ring adopts a twist conformation. The three F atoms of the CF_3_ group are disordered over two sets of sites with refined occupancies of 0.542 (18) and 0.458 (18). In the nine-membered 1*H*-indoline ring system, the 1*H*-pyrrole ring forms a dihedral angle of 4.7 (2)° with the benzene ring, while it is twisted at an angle of 46.5 (2)° with respect to the attached phenyl ring. The dihedral angle between the phenyl and trifluoro­methyl-substituted benzene rings is 56.0 (2)°. In the crystal, N—H⋯O hydrogen bonds connect the mol­ecules into a three-dimensional network. In addition, weak C—H⋯O hydrogen bonds and weak C—H⋯π inter­actions are observed.

## Related literature

For medicinal applications of indole derivatives, see: Beale (2011 ▶); Brancale & Silvestri (2007 ▶); Cihan-Ustundag & Capan (2012 ▶); Oudard *et al.* (2011 ▶); Verma & Saraf (2008 ▶). For the definition of ring-puckering parameters, see: Cremer & Pople (1975 ▶). For related structures, see: Akkurt *et al.* (2010 ▶, 2011*a* ▶,*b* ▶,*c* ▶). For standard values of bond lengths, see: Allen *et al.* (1987 ▶).
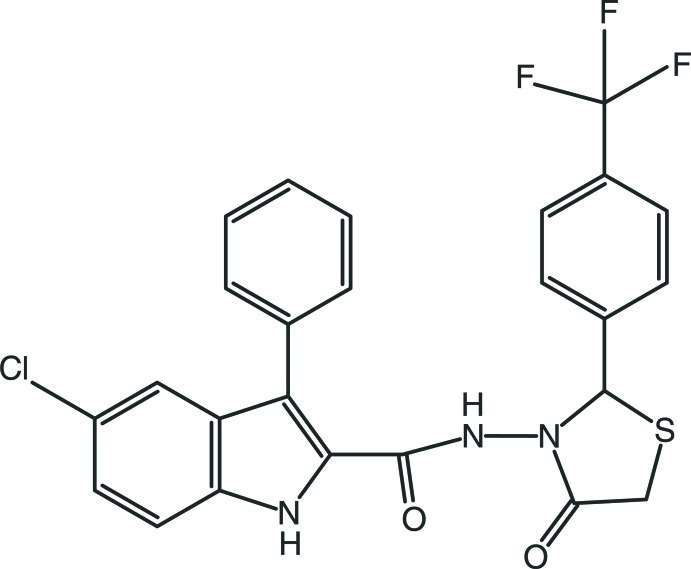


## Experimental

### 

#### Crystal data

C_25_H_17_ClF_3_N_3_O_2_S
*M* *_r_* = 515.94
Tetragonal, 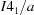

*a* = 22.9020 (6) Å
*c* = 18.3260 (6) Å
*V* = 9612.0 (6) Å^3^
*Z* = 16
Mo *K*α radiation
μ = 0.30 mm^−1^
*T* = 296 K
0.57 × 0.43 × 0.29 mm

#### Data collection

Stoe IPDS 2 diffractometer
Absorption correction: integration (*X-RED32*; Stoe & Cie, 2002 ▶) *T*_min_ = 0.849, *T*_max_ = 0.919
71781 measured reflections
4968 independent reflections
3054 reflections with *I* > 2σ(*I*)
*R*_int_ = 0.076

#### Refinement

*R*[*F* ^2^ > 2σ(*F* ^2^)] = 0.073
*wR*(*F* ^2^) = 0.210
*S* = 1.05
4968 reflections
345 parameters
14 restraints
H-atom parameters constrained
Δρ_max_ = 0.53 e Å^−3^
Δρ_min_ = −0.30 e Å^−3^

### 

Data collection: *X-AREA* (Stoe & Cie, 2002 ▶); cell refinement: *X-AREA*; data reduction: *X-RED32* (Stoe & Cie, 2002 ▶); program(s) used to solve structure: *SIR97* (Altomare *et al.*, 1999 ▶); program(s) used to refine structure: *SHELXL97* (Sheldrick, 2008 ▶); molecular graphics: *ORTEP-3* (Farrugia, 1997 ▶); software used to prepare material for publication: *WinGX* (Farrugia, 1999 ▶).

## Supplementary Material

Crystal structure: contains datablock(s) global, I. DOI: 10.1107/S1600536812039347/lh5532sup1.cif

Structure factors: contains datablock(s) I. DOI: 10.1107/S1600536812039347/lh5532Isup2.hkl

Supplementary material file. DOI: 10.1107/S1600536812039347/lh5532Isup3.cml

Additional supplementary materials:  crystallographic information; 3D view; checkCIF report

## Figures and Tables

**Table 1:** Hydrogen-bond geometry (Å, °) *Cg*1 and *Cg*2 are the centroids of the N1/C1/C6–C8 and C9–C14 rings, respectively.

*D*—H⋯*A*	*D*—H	H⋯*A*	*D*⋯*A*	*D*—H⋯*A*
N1—H1⋯O1^i^	0.86	2.08	2.864 (4)	151
N2—H2*A*⋯O2^ii^	0.86	2.34	2.851 (4)	118
C18—H18⋯O2^ii^	0.98	2.53	3.145 (5)	121
C24—H24⋯*Cg*1^iii^	0.93	2.77	3.438 (5)	129
C17—H17*B*⋯*Cg*2^iv^	0.97	2.95	3.799 (5)	147

Symmetry codes: (i) 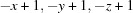; (ii) 
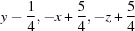
; (iii) 
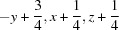
; (iv) 
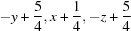
.

**Fig. 1.**
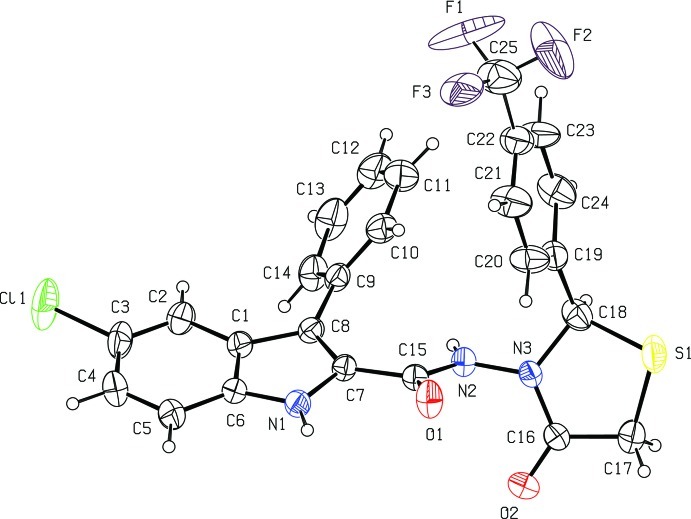
The molecular structure of the title compound with displacement ellipsoids for non-H atoms drawn at the 30% probability level. The disorder is not shown.

## supplementary crystallographic information

### Comment

The indole ring system is one of the most encountered heterocyles in natural and synthetic drug compounds (Brancale & Silvestri, 2007). Several indole derivatives, such as sunitinib as tyrosine kinase inhibitor (Oudard *et al.*, 2011) or delavirdine as nonnucleoside reverse transcriptase inhibitor (Beale, 2011), are in clinical use. Here, we aimed to combine this basic scaffold with 4-thiazolidinones, which have been shown to have anticancer (Cihan-Ustundag & Capan, 2012), antifungal, antibacterial and antiviral properties (Verma & Saraf, 2008), to obtain leads toward the design of anticancer compounds.

The molecular structure of the title compound is shown in Fig. 1. The 1,3-thiazolidine ring (S1/N3/C16–C18) has a twist conformation as indicated by the puckering parameters (Cremer & Pople, 1975) of Q(2) = 0.354 (4) Å, φ(2) = 342.0 (6)°. In the nine-membered 1H-indoline ring system (N1/C1–C8), the 1*H*-pyrrole ring (N1C1/C6–C8) makes a dihedral angle of 4.7 (2)° with the benzene ring (C1–C6), while it is twisted an angle of 46.5 (2)° with respect to the attached phenyl ring (C9–C14). The dihedral angle between the C9–C14 phenyl and C19–C24 benzene rings which are attached to the *H*-pyrrole and 1,3-thiazolidine rings, respectively, is 56.0 (2)°.

All bond lengths and bond angles in (I) are within normal ranges (Allen *et al.*, 1987) and are comparable to those reported for the related structures (Akkurt *et al.*, 2010, 2011*a*,*b*,*c*). The C7—C15—N2—N3, N1—C7—C15—O1 and O1—C15—N2—N3 torsion angles are 176.4 (3), -19.5 (5) and -2.6 (5)°, respectively.

In the crystal, N—H···O and weak C—H···O hydrogen bonds, and C—H···π interactions stabilize the crystal structure, forming a three-dimensional network (Table 1).

### Experimental

0.002 mol of *N*'-(4-(trifluoromethyl)benzylidene-5-chloro-3-phenyl-*1H-*indole-2-carbohydrazide was reacted with 3 ml of mercaptoacetic acid in anhydrous benzene for 6 h using a Dean–Stark trap. Excess benzene was removed under reduced pressure. The residue was triturated with saturated sodium bicarbonate solution. The separated solid was filtered, washed with water and crystallized from ethanol. Orange crystalline solid. m.p. 510 K. IR (KBr) ν 3303 (indol N—H and amide N—H); 1717 (C═O),1655, (C═O) cm^-1^; Analysis calculated for C_25_H_17_ClF_3_N_3_O_2_S: C: 58.20; H: 3.32; N: 8.14%. Found: C: 57.18; H: 3.59; N: 7.87%.

### Refinement

H atoms were positioned geometrically, with N—H = 0.86 Å, C—H = 0.93 Å (aromatic), 0.97 Å (methylene) and 0.98 Å (methine) H atoms, respectively, and refined as riding with *U*_iso_(H) = 1.2 *U*_eq_(C, N). The three fluorine atoms of the CF_3_ group are disordered over two sets of sites in a 0.542 (18):0.458 (18) ratio. The carbon atom of the CF_3_ group was refined as two atoms (C25 and C25') sharing the same site [their xyz and *U^ij^* parameters were equated by dummy atom constraints using the EXYZ and EADP commands]. Thirteen poorly fitted reflections (-4 6 0), (-2 4 2), (2 8 2), (-3 6 1), (0 6 2), (2 9 5), (-1 3 6), (1 1 2), (-1 2 3), (1 2 3), (2 2 2), (-7 10 7) and (3 4 3) were omitted from the refinement. These reflections have Fobs much greater than Fcalc and this might be attributed to the poor quality of the available crystal and the presence of disorder in the structure.

### Crystal data

**Table:**

C_25_H_17_ClF_3_N_3_O_2_S	*D*_x_ = 1.426 Mg m^−^^3^
*M**_r_* = 515.94	Mo *K*α radiation, λ = 0.71073 Å
Tetragonal, *I*4_1_/*a*	Cell parameters from 45035 reflections
Hall symbol: -I 4ad	θ = 1.4–27.8°
*a* = 22.9020 (6) Å	µ = 0.30 mm^−^^1^
*c* = 18.3260 (6) Å	*T* = 296 K
*V* = 9612.0 (6) Å^3^	Prism, orange
*Z* = 16	0.57 × 0.43 × 0.29 mm
*F*(000) = 4224	

### Data collection

**Table:**

Stoe IPDS 2 diffractometer	4968 independent reflections
Radiation source: sealed X-ray tube, 12 x 0.4 mm long-fine focus	3054 reflections with *I* > 2σ(*I*)
Plane graphite monochromator	*R*_int_ = 0.076
Detector resolution: 6.67 pixels mm^-1^	θ_max_ = 26.5°, θ_min_ = 1.8°
ω scans	*h* = −28→28
Absorption correction: integration (*X-RED32*; Stoe & Cie, 2002)	*k* = −28→28
*T*_min_ = 0.849, *T*_max_ = 0.919	*l* = −22→22
71781 measured reflections	

### Refinement

**Table:**

Refinement on *F*^2^	Primary atom site location: structure-invariant direct methods
Least-squares matrix: full	Secondary atom site location: difference Fourier map
*R*[*F*^2^ > 2σ(*F*^2^)] = 0.073	Hydrogen site location: inferred from neighbouring sites
*wR*(*F*^2^) = 0.210	H-atom parameters constrained
*S* = 1.05	*w* = 1/[σ^2^(*F*_o_^2^) + (0.1132*P*)^2^] where *P* = (*F*_o_^2^ + 2*F*_c_^2^)/3
4968 reflections	(Δ/σ)_max_ < 0.001
345 parameters	Δρ_max_ = 0.53 e Å^−^^3^
14 restraints	Δρ_min_ = −0.30 e Å^−^^3^

### Special details

**Table:**

Experimental. ^1^H-NMR (400 MHz) (DMSO d6/TMS) δ 3.80 (1*H*, d, J = 15.90 Hz, H5-thia.), 3.96 (1*H*, dd, J1 = 15.87 Hz, J2 = 1.71 Hz, H5-thia.), 5.97 (1*H*, s, H2-thia.), 7.19–7.21 (3*H*, m, 3-C_6_H_5_-ind.), 7.25–7.29 (3*H*, m, H6-ind, 3-C_6_H_5_-ind.) 7.43–7.51 (2H,m H4-, H7-ind.), 7.67 (2*H*, d, J = 8.23 Hz, 2-C_6_H_4_-(H2,6)-thia), 7.75 (2*H*, d, J = 8.31 Hz, 2-C_6_H_4_-(H3,5)-thia.), 10.19 (1*H*, s, CONH), 12.04 (1*H*, s, NH) p.p.m.; ^13^C-NMR (HMBC) (125 MHz) (DMSO-d6) δ 29.91 (C5-thia.), 61.66 (C2-thia.), 114.92 (C7-ind.), 119.24 (C3-ind.), 119.68 (C4-ind.) 125.13 (C6-ind.), 125.84 (C3a-ind), 126.25 (2-C_6_H_4_-(C3,5)-thia.), 127.18 (C5-ind.), 127.93 (C2-ind.), 128.92 (2-C_6_H_4_-(C2,6)-thia.), 133.06 (3-C_6_H_5_-(C1)-ind), 134.99 (C7a-ind), 144.17 (2-C_6_-H_4_-(C1)-thia.), 161.21 (CONH), 169.66 (C═O) p.p.m.; APCI+ m/*z* (%) = 516.1/518.2 (MH^+^, 29.4/12.7), 79.5 (100).
Geometry. Bond distances, angles *etc*. have been calculated using the rounded fractional coordinates. All su's are estimated from the variances of the (full) variance-covariance matrix. The cell e.s.d.'s are taken into account in the estimation of distances, angles and torsion angles
Refinement. Refinement on *F*^2^ for ALL reflections except those flagged by the user for potential systematic errors. Weighted *R*-factors *wR* and all goodnesses of fit *S* are based on *F*^2^, conventional *R*-factors *R* are based on *F*, with *F* set to zero for negative *F*^2^. The observed criterion of *F*^2^ > σ(*F*^2^) is used only for calculating -*R*-factor-obs *etc*. and is not relevant to the choice of reflections for refinement. *R*-factors based on *F*^2^ are statistically about twice as large as those based on *F*, and *R*-factors based on ALL data will be even larger.

### Fractional atomic coordinates and isotropic or equivalent isotropic displacement parameters (Å^2^)

**Table:**

	*x*	*y*	*z*	*U*_iso_*/*U*_eq_	Occ. (<1)
Cl1	0.52469 (9)	0.62509 (8)	0.11058 (7)	0.1214 (8)	
S1	0.33741 (6)	0.62264 (6)	0.73456 (6)	0.0809 (5)	
O1	0.44994 (13)	0.54850 (11)	0.54646 (14)	0.0633 (10)	
O2	0.49070 (12)	0.64975 (16)	0.66104 (17)	0.0816 (13)	
N1	0.50355 (13)	0.56361 (13)	0.41587 (15)	0.0493 (9)	
N2	0.41564 (12)	0.64048 (12)	0.54712 (15)	0.0455 (9)	
N3	0.39991 (12)	0.63564 (12)	0.61935 (14)	0.0439 (9)	
C1	0.48360 (15)	0.62222 (16)	0.32162 (18)	0.0495 (11)	
C2	0.48633 (19)	0.63894 (18)	0.2484 (2)	0.0623 (14)	
C3	0.5236 (2)	0.6087 (2)	0.2038 (2)	0.0733 (16)	
C4	0.5597 (2)	0.5641 (2)	0.2289 (2)	0.0763 (19)	
C5	0.55736 (18)	0.5467 (2)	0.2994 (2)	0.0647 (14)	
C6	0.51797 (15)	0.57519 (16)	0.34551 (18)	0.0481 (11)	
C7	0.46139 (15)	0.60221 (14)	0.43855 (17)	0.0439 (11)	
C8	0.44742 (15)	0.63943 (15)	0.38228 (18)	0.0464 (11)	
C9	0.39990 (17)	0.68331 (16)	0.37637 (18)	0.0512 (11)	
C10	0.34426 (19)	0.6712 (2)	0.3996 (2)	0.0657 (16)	
C11	0.2991 (2)	0.7108 (3)	0.3882 (3)	0.0867 (19)	
C12	0.3093 (3)	0.7615 (3)	0.3509 (3)	0.092 (2)	
C13	0.3642 (3)	0.7747 (2)	0.3279 (2)	0.087 (2)	
C14	0.4097 (2)	0.73647 (18)	0.3411 (2)	0.0683 (14)	
C15	0.44188 (15)	0.59419 (14)	0.51464 (18)	0.0446 (11)	
C16	0.43981 (16)	0.64471 (17)	0.6722 (2)	0.0523 (12)	
C17	0.41203 (19)	0.6458 (2)	0.7458 (2)	0.0737 (16)	
C18	0.33692 (18)	0.64343 (19)	0.6371 (2)	0.0647 (14)	
C19	0.29649 (17)	0.60608 (18)	0.5938 (2)	0.0587 (14)	
C20	0.3035 (2)	0.54741 (18)	0.5819 (3)	0.0733 (17)	
C21	0.2629 (2)	0.5169 (2)	0.5429 (3)	0.0863 (19)	
C22	0.21510 (19)	0.5420 (2)	0.5160 (3)	0.0747 (17)	
C23	0.2074 (2)	0.5992 (3)	0.5246 (3)	0.092 (2)	
C24	0.2472 (2)	0.6329 (2)	0.5623 (3)	0.0813 (18)	
C25	0.1692 (3)	0.5063 (3)	0.4760 (4)	0.111 (3)	0.500
C25'	0.1692 (3)	0.5063 (3)	0.4760 (4)	0.111 (3)	0.500
F1	0.1487 (8)	0.5287 (4)	0.4197 (9)	0.197 (8)	0.542 (18)
F2	0.1278 (6)	0.4936 (8)	0.5184 (9)	0.252 (12)	0.542 (18)
F3	0.1890 (4)	0.4577 (4)	0.4534 (5)	0.108 (4)	0.542 (18)
F1'	0.1752 (7)	0.5149 (15)	0.4092 (6)	0.37 (2)	0.458 (18)
F2'	0.1190 (6)	0.5316 (11)	0.4860 (11)	0.227 (12)	0.458 (18)
F3'	0.1645 (14)	0.4561 (7)	0.494 (2)	0.42 (2)	0.458 (18)
H1	0.51850	0.53630	0.44220	0.0590*	
H2	0.46370	0.66950	0.23060	0.0750*	
H2A	0.40900	0.67210	0.52320	0.0550*	
H4	0.58570	0.54610	0.19690	0.0920*	
H5	0.58120	0.51680	0.31660	0.0780*	
H10	0.33660	0.63610	0.42340	0.0790*	
H11	0.26190	0.70280	0.40590	0.1040*	
H12	0.27870	0.78700	0.34120	0.1100*	
H13	0.37110	0.80950	0.30330	0.1040*	
H14	0.44730	0.74630	0.32620	0.0820*	
H17A	0.41340	0.68490	0.76600	0.0880*	
H17B	0.43240	0.61960	0.77870	0.0880*	
H18	0.32600	0.68460	0.63200	0.0770*	
H20	0.33610	0.52830	0.60040	0.0880*	
H21	0.26890	0.47720	0.53490	0.1030*	
H23	0.17450	0.61690	0.50470	0.1100*	
H24	0.24160	0.67300	0.56690	0.0970*	

### Atomic displacement parameters (Å^2^)

**Table:**

	*U*^11^	*U*^22^	*U*^33^	*U*^12^	*U*^13^	*U*^23^
Cl1	0.1764 (17)	0.1380 (14)	0.0499 (6)	0.0150 (11)	0.0362 (8)	0.0157 (7)
S1	0.0795 (8)	0.1053 (10)	0.0579 (6)	−0.0299 (7)	0.0263 (5)	−0.0225 (6)
O1	0.091 (2)	0.0421 (14)	0.0567 (15)	0.0209 (13)	0.0195 (14)	0.0083 (12)
O2	0.0434 (17)	0.129 (3)	0.0724 (19)	0.0023 (17)	−0.0013 (14)	−0.0113 (18)
N1	0.0518 (17)	0.0503 (17)	0.0459 (15)	0.0110 (14)	0.0002 (13)	−0.0016 (13)
N2	0.0564 (17)	0.0372 (15)	0.0430 (14)	0.0060 (13)	0.0052 (13)	0.0036 (12)
N3	0.0420 (16)	0.0464 (16)	0.0434 (15)	0.0043 (12)	0.0070 (12)	−0.0025 (12)
C1	0.047 (2)	0.057 (2)	0.0446 (18)	−0.0042 (17)	0.0022 (15)	0.0004 (16)
C2	0.075 (3)	0.062 (2)	0.050 (2)	−0.001 (2)	0.0029 (19)	0.0076 (18)
C3	0.090 (3)	0.088 (3)	0.042 (2)	−0.008 (3)	0.017 (2)	0.004 (2)
C4	0.072 (3)	0.097 (4)	0.060 (3)	0.006 (3)	0.021 (2)	−0.011 (2)
C5	0.059 (2)	0.078 (3)	0.057 (2)	0.013 (2)	0.0061 (19)	−0.007 (2)
C6	0.0431 (19)	0.059 (2)	0.0422 (17)	0.0002 (16)	0.0001 (14)	−0.0034 (16)
C7	0.049 (2)	0.0433 (18)	0.0395 (17)	0.0042 (15)	0.0011 (14)	−0.0007 (14)
C8	0.049 (2)	0.048 (2)	0.0423 (17)	0.0019 (16)	−0.0036 (15)	0.0008 (15)
C9	0.060 (2)	0.055 (2)	0.0385 (17)	0.0102 (18)	−0.0020 (16)	0.0012 (16)
C10	0.068 (3)	0.072 (3)	0.057 (2)	0.014 (2)	−0.007 (2)	0.007 (2)
C11	0.072 (3)	0.110 (4)	0.078 (3)	0.034 (3)	−0.007 (2)	0.009 (3)
C12	0.110 (4)	0.097 (4)	0.068 (3)	0.057 (3)	−0.018 (3)	0.005 (3)
C13	0.138 (5)	0.062 (3)	0.061 (3)	0.038 (3)	0.000 (3)	0.014 (2)
C14	0.090 (3)	0.059 (2)	0.056 (2)	0.017 (2)	0.008 (2)	0.0124 (19)
C15	0.0468 (19)	0.0401 (19)	0.0469 (18)	0.0047 (15)	0.0000 (15)	0.0019 (15)
C16	0.045 (2)	0.060 (2)	0.052 (2)	0.0065 (17)	0.0041 (17)	−0.0046 (17)
C17	0.074 (3)	0.094 (3)	0.053 (2)	−0.003 (2)	0.006 (2)	−0.013 (2)
C18	0.056 (2)	0.059 (2)	0.079 (3)	−0.0005 (19)	0.010 (2)	−0.011 (2)
C19	0.053 (2)	0.064 (3)	0.059 (2)	−0.016 (2)	0.0066 (18)	−0.0145 (18)
C20	0.062 (3)	0.056 (3)	0.102 (3)	0.012 (2)	−0.022 (2)	−0.004 (2)
C21	0.071 (3)	0.062 (3)	0.126 (4)	0.003 (2)	−0.016 (3)	−0.032 (3)
C22	0.058 (3)	0.083 (3)	0.083 (3)	0.009 (2)	−0.009 (2)	−0.023 (2)
C23	0.061 (3)	0.094 (4)	0.120 (4)	0.015 (3)	−0.034 (3)	−0.004 (3)
C24	0.071 (3)	0.052 (2)	0.121 (4)	0.017 (2)	0.006 (3)	−0.007 (3)
C25	0.085 (4)	0.106 (5)	0.141 (6)	0.004 (4)	−0.020 (4)	−0.041 (5)
C25'	0.085 (4)	0.106 (5)	0.141 (6)	0.004 (4)	−0.020 (4)	−0.041 (5)
F1	0.231 (16)	0.109 (7)	0.252 (17)	0.000 (6)	−0.205 (15)	−0.005 (7)
F2	0.110 (9)	0.37 (3)	0.275 (18)	−0.148 (12)	0.098 (11)	−0.244 (19)
F3	0.098 (5)	0.106 (7)	0.120 (7)	−0.009 (4)	−0.037 (5)	−0.063 (5)
F1'	0.112 (11)	0.88 (7)	0.105 (11)	−0.010 (19)	−0.014 (8)	−0.20 (2)
F2'	0.070 (7)	0.40 (3)	0.210 (18)	0.013 (12)	−0.056 (10)	−0.102 (18)
F3'	0.54 (5)	0.151 (18)	0.57 (5)	−0.16 (2)	−0.42 (4)	0.14 (3)

### Geometric parameters (Å, º)

**Table:**

Cl1—C3	1.749 (4)	C12—C13	1.360 (9)
S1—C17	1.801 (5)	C13—C14	1.382 (7)
S1—C18	1.849 (4)	C16—C17	1.492 (5)
O1—C15	1.212 (4)	C18—C19	1.490 (6)
O2—C16	1.189 (5)	C19—C20	1.371 (6)
N1—C6	1.357 (4)	C19—C24	1.409 (6)
N1—C7	1.374 (4)	C20—C21	1.365 (7)
N2—N3	1.376 (4)	C21—C22	1.331 (7)
N2—C15	1.356 (4)	C22—C23	1.331 (8)
N3—C16	1.348 (5)	C22—C25	1.520 (8)
N3—C18	1.490 (5)	C22—C25'	1.520 (8)
N1—H1	0.8600	C23—C24	1.380 (7)
N2—H2A	0.8600	C2—H2	0.9300
C1—C2	1.397 (5)	C4—H4	0.9300
C1—C6	1.404 (5)	C5—H5	0.9300
C1—C8	1.441 (5)	C10—H10	0.9300
C2—C3	1.370 (6)	C11—H11	0.9300
C3—C4	1.392 (6)	C12—H12	0.9300
C4—C5	1.353 (5)	C13—H13	0.9300
C5—C6	1.398 (5)	C14—H14	0.9300
C7—C8	1.376 (5)	C17—H17A	0.9700
C7—C15	1.476 (5)	C17—H17B	0.9700
C8—C9	1.485 (5)	C18—H18	0.9800
C9—C10	1.372 (6)	C20—H20	0.9300
C9—C14	1.397 (5)	C21—H21	0.9300
C10—C11	1.391 (7)	C23—H23	0.9300
C11—C12	1.368 (9)	C24—H24	0.9300
			
C17—S1—C18	92.32 (18)	S1—C18—C19	111.8 (3)
C6—N1—C7	109.4 (3)	C18—C19—C24	117.8 (4)
N3—N2—C15	118.4 (3)	C20—C19—C24	117.1 (4)
N2—N3—C16	120.1 (3)	C18—C19—C20	125.1 (4)
N2—N3—C18	117.0 (3)	C19—C20—C21	120.4 (4)
C16—N3—C18	118.8 (3)	C20—C21—C22	122.2 (4)
C7—N1—H1	125.00	C23—C22—C25'	119.7 (5)
C6—N1—H1	125.00	C21—C22—C25	121.0 (5)
N3—N2—H2A	121.00	C21—C22—C25'	121.0 (5)
C15—N2—H2A	121.00	C21—C22—C23	119.4 (5)
C2—C1—C6	119.0 (3)	C23—C22—C25	119.7 (5)
C2—C1—C8	133.8 (3)	C22—C23—C24	121.5 (5)
C6—C1—C8	107.0 (3)	C19—C24—C23	119.4 (4)
C1—C2—C3	117.6 (4)	C1—C2—H2	121.00
C2—C3—C4	122.9 (4)	C3—C2—H2	121.00
Cl1—C3—C2	118.9 (3)	C3—C4—H4	120.00
Cl1—C3—C4	118.1 (3)	C5—C4—H4	120.00
C3—C4—C5	120.5 (4)	C4—C5—H5	121.00
C4—C5—C6	117.7 (4)	C6—C5—H5	121.00
N1—C6—C1	108.0 (3)	C9—C10—H10	120.00
N1—C6—C5	129.8 (3)	C11—C10—H10	120.00
C1—C6—C5	122.1 (3)	C10—C11—H11	120.00
N1—C7—C8	109.6 (3)	C12—C11—H11	120.00
C8—C7—C15	135.7 (3)	C11—C12—H12	120.00
N1—C7—C15	114.8 (3)	C13—C12—H12	120.00
C1—C8—C7	106.0 (3)	C12—C13—H13	120.00
C1—C8—C9	123.4 (3)	C14—C13—H13	120.00
C7—C8—C9	130.1 (3)	C9—C14—H14	120.00
C10—C9—C14	118.0 (4)	C13—C14—H14	120.00
C8—C9—C14	120.4 (3)	S1—C17—H17A	110.00
C8—C9—C10	121.4 (3)	S1—C17—H17B	110.00
C9—C10—C11	120.8 (4)	C16—C17—H17A	110.00
C10—C11—C12	120.1 (5)	C16—C17—H17B	110.00
C11—C12—C13	120.1 (6)	H17A—C17—H17B	109.00
C12—C13—C14	120.1 (5)	S1—C18—H18	110.00
C9—C14—C13	120.8 (4)	N3—C18—H18	110.00
O1—C15—N2	122.1 (3)	C19—C18—H18	110.00
O1—C15—C7	121.1 (3)	C19—C20—H20	120.00
N2—C15—C7	116.9 (3)	C21—C20—H20	120.00
O2—C16—C17	124.9 (4)	C20—C21—H21	119.00
N3—C16—C17	111.3 (3)	C22—C21—H21	119.00
O2—C16—N3	123.8 (3)	C22—C23—H23	119.00
S1—C17—C16	107.2 (3)	C24—C23—H23	119.00
N3—C18—C19	114.6 (3)	C19—C24—H24	120.00
S1—C18—N3	100.0 (2)	C23—C24—H24	120.00
			
C17—S1—C18—C19	150.1 (3)	C15—C7—C8—C1	−179.5 (4)
C18—S1—C17—C16	−22.9 (3)	C15—C7—C8—C9	−8.0 (7)
C17—S1—C18—N3	28.4 (3)	N1—C7—C8—C1	−0.1 (4)
C6—N1—C7—C15	−180.0 (3)	N1—C7—C8—C9	171.4 (3)
C7—N1—C6—C1	−0.8 (4)	C8—C7—C15—O1	159.8 (4)
C6—N1—C7—C8	0.5 (4)	C8—C7—C15—N2	−21.3 (6)
C7—N1—C6—C5	−178.2 (4)	N1—C7—C15—N2	159.4 (3)
N3—N2—C15—O1	2.6 (5)	C7—C8—C9—C14	142.6 (4)
C15—N2—N3—C16	82.3 (4)	C7—C8—C9—C10	−43.0 (6)
C15—N2—N3—C18	−120.9 (3)	C1—C8—C9—C10	127.2 (4)
N3—N2—C15—C7	−176.4 (3)	C1—C8—C9—C14	−47.2 (5)
C18—N3—C16—C17	15.4 (5)	C8—C9—C10—C11	−174.6 (4)
N2—N3—C18—C19	52.5 (4)	C8—C9—C14—C13	172.5 (3)
N2—N3—C16—C17	171.8 (3)	C10—C9—C14—C13	−2.2 (5)
C16—N3—C18—C19	−150.3 (3)	C14—C9—C10—C11	0.0 (6)
C16—N3—C18—S1	−30.6 (4)	C9—C10—C11—C12	2.6 (7)
C18—N3—C16—O2	−166.3 (4)	C10—C11—C12—C13	−3.2 (8)
N2—N3—C18—S1	172.2 (2)	C11—C12—C13—C14	1.1 (8)
N2—N3—C16—O2	−9.8 (6)	C12—C13—C14—C9	1.7 (6)
C8—C1—C2—C3	−173.9 (4)	O2—C16—C17—S1	−169.2 (4)
C6—C1—C2—C3	−1.1 (6)	N3—C16—C17—S1	9.1 (4)
C6—C1—C8—C7	−0.4 (4)	S1—C18—C19—C20	−65.7 (5)
C2—C1—C6—N1	−173.9 (3)	S1—C18—C19—C24	114.5 (4)
C2—C1—C6—C5	3.8 (6)	N3—C18—C19—C20	47.2 (5)
C2—C1—C8—C9	0.9 (6)	N3—C18—C19—C24	−132.6 (4)
C2—C1—C8—C7	173.1 (4)	C18—C19—C20—C21	178.2 (4)
C6—C1—C8—C9	−172.6 (3)	C24—C19—C20—C21	−2.0 (7)
C8—C1—C6—C5	178.4 (3)	C18—C19—C24—C23	−177.1 (4)
C8—C1—C6—N1	0.7 (4)	C20—C19—C24—C23	3.1 (7)
C1—C2—C3—Cl1	175.8 (3)	C19—C20—C21—C22	−1.0 (8)
C1—C2—C3—C4	−2.3 (7)	C20—C21—C22—C23	2.8 (8)
Cl1—C3—C4—C5	−175.0 (4)	C20—C21—C22—C25	−177.0 (5)
C2—C3—C4—C5	3.1 (7)	C20—C21—C22—C25'	−177.0 (5)
C3—C4—C5—C6	−0.4 (6)	C21—C22—C23—C24	−1.6 (8)
C4—C5—C6—N1	174.1 (4)	C25—C22—C23—C24	178.2 (5)
C4—C5—C6—C1	−3.0 (6)	C25'—C22—C23—C24	178.2 (5)
N1—C7—C15—O1	−19.5 (5)	C22—C23—C24—C19	−1.4 (8)

### Hydrogen-bond geometry (Å, º)

Cg1 and Cg2 are the centroids of the N1/C1/C6–C8 and C9–C14 rings, respectively.

**Table:**

*D*—H···*A*	*D*—H	H···*A*	*D*···*A*	*D*—H···*A*
N1—H1···O1^i^	0.86	2.08	2.864 (4)	151
N2—H2*A*···O2^ii^	0.86	2.34	2.851 (4)	118
C18—H18···O2^ii^	0.98	2.53	3.145 (5)	121
C21—H21···F3	0.93	2.40	2.719 (10)	100
C24—H24···*Cg*1^iii^	0.93	2.77	3.438 (5)	129
C17—H17*B*···*Cg*2^iv^	0.97	2.95	3.799 (5)	147

Symmetry codes: (i) −*x*+1, −*y*+1, −*z*+1; (ii) *y*−1/4, −*x*+5/4, −*z*+5/4; (iii) −*y*+3/4, *x*+1/4, *z*+1/4; (iv) −*y*+5/4, *x*+1/4, −*z*+5/4.

## References

[bb1] Akkurt, M., Çelik, İ., Demir, H., Özkırımlı, S. & Büyükgüngör, O. (2010). *Acta Cryst.* E**66**, o1691–o1692. 10.1107/S1600536810022506 PMC3006680 21587914

[bb2] Akkurt, M., Çelik, İ., Demir, H., Özkırımlı, S. & Büyükgüngör, O. (2011*a*). *Acta Cryst.* E**67**, o293–o294. 10.1107/S1600536811000481 PMC3051725 21522985

[bb3] Akkurt, M., Çelik, İ., Demir, H., Özkırımlı, S. & Büyükgüngör, O. (2011*b*). *Acta Cryst.* E**67**, o745–o746. 10.1107/S1600536811007136 PMC3099898 21754043

[bb4] Akkurt, M., Çelik, İ., Demir, H., Özkırımlı, S. & Büyükgüngör, O. (2011*c*). *Acta Cryst.* E**67**, o914–o915. 10.1107/S1600536811009603 PMC3100075 21754186

[bb5] Allen, F. H., Kennard, O., Watson, D. G., Brammer, L., Orpen, A. G. & Taylor, R. (1987). *J. Chem. Soc. Perkin Trans. 2*, pp. S1–19.

[bb6] Altomare, A., Burla, M. C., Camalli, M., Cascarano, G. L., Giacovazzo, C., Guagliardi, A., Moliterni, A. G. G., Polidori, G. & Spagna, R. (1999). *J. Appl. Cryst.* **32**, 115–119.

[bb7] Beale, J. M. (2011). *Wilson and Gisvold’s Textbook of Organic Medicinal and Pharmaceutical Chemistry*, 12th ed., edited by J. M. Beale & J. H. Block, pp. 342–352. Philadelphia: Lippincott Williams and Wilkins.

[bb8] Brancale, A. & Silvestri, R. (2007). *Med. Res. Rev.* **27**, 209–238. 10.1002/med.20080 16788980

[bb9] Cihan-Ustundag, G. & Capan, G. (2012). *Mol Divers* doi:10.1007/s11030-012-9385-y. 10.1007/s11030-012-9385-y 22893206

[bb10] Cremer, D. & Pople, J. A. (1975). *J. Am. Chem. Soc.* **97**, 1354–1358.

[bb11] Farrugia, L. J. (1997). *J. Appl. Cryst.* **30**, 565.

[bb12] Farrugia, L. J. (1999). *J. Appl. Cryst.* **32**, 837–838.

[bb13] Oudard, S., Beuselinck, B., Decoene, J. & Albers, P. (2011). *Cancer Treat. Rev.* **37**, 178–184. 10.1016/j.ctrv.2010.08.005 20817406

[bb14] Sheldrick, G. M. (2008). *Acta Cryst.* A**64**, 112–122. 10.1107/S0108767307043930 18156677

[bb15] Stoe & Cie (2002). *X-AREA* and *X-RED32* Stoe & Cie, Darmstadt, Germany.

[bb16] Verma, A. & Saraf, K. S. (2008). *Eur. J. Med. Chem.* **43**, 897–905. 10.1016/j.ejmech.2007.07.017 17870209

